# Additives with Emerging Health Concerns in Ultra-Processed Sweetened Beverages Sold in the United States: Preservatives, Artificial Sweeteners, and Added Sugars

**DOI:** 10.3390/nu18132176

**Published:** 2026-07-04

**Authors:** Elizabeth K. Dunford, Mona S. Calvo, Jaime Uribarri

**Affiliations:** 1 The George Institute for Global Health, University of New South Wales, Sydney, NSW 2031, Australia; 2 Department of Nutrition, Gillings Global School of Public Health, The University of North Carolina at Chapel Hill, Chapel Hill, NC 27599, USA; 3 Department of Medicine, Division of Nephrology, Icahn School of Medicine at Mount Sinai, New York, NY 10029, USA; mscalvo55@comcast.net (M.S.C.); jaime.uribarri@mountsinai.org (J.U.)

**Keywords:** non-nutritive sweeteners, phosphoric acid, phosphate-additives, sorbates, benzoates, non-communicable disease risk, ultra-processed food and drinks

## Abstract

Background: Consumption of ultra-processed foods (UPFs) continues to rise alongside a growing body of epidemiological evidence linking high UPF intake to adverse health outcomes, including cardiovascular disease and type 2 diabetes, in the general population. However, the factors underlying these associations remain incompletely understood, underscoring the need to examine components beyond traditional nutrient composition. In particular, food-processing additives are increasingly recognized as defining features of industrially formulated UPFs. Objective/Methods: In this study, we used a large food label database to cross-sectionally examine the presence and co-occurrence of selected additives (sorbates, benzoates, phosphate additives, and non-nutritive sweeteners [NNSs]) in sweetened beverages sold by the 25 top-selling U.S. food and beverage manufacturers in 2020. Results: We found that sweetened beverages marketed in the U.S. frequently contain multiple additive classes concurrently, supporting the concept that these products represent complex chemical exposure mixtures rather than simple combinations of water and sweeteners. Formulations containing multiple additives were substantially more common than simpler formulations, with many beverages simultaneously containing combinations of sweeteners, preservatives, and phosphate additives. Products containing NNS exhibited higher additive clustering compared to products containing added sugar. Conclusions: Collectively, these findings support the need for broader consideration of beverage formulation complexity in nutrition research, dietary guidance, and policy regulation.

## 1. Introduction

Consumption of ultra-processed food and beverages (UPFs) continues to rise alongside a growing body of epidemiological evidence linking high UPF intake to chronic disease risk in the global population [1]. The extent of modern industrial food processing has increasingly overtaken traditional nutrient composition as a key factor evaluating a food’s healthfulness and its association with chronic disease risk. Importantly, this relationship between level of processing and disease risk appears to hold regardless of the classification system used to assess the degree of processing [2]. Nonetheless, the factors underlying these associations remain unclear, underscoring the need to examine factors beyond UPF nutrient adequacy and explore ingredients that identify foods as UPF, specifically food additives [3,4,5]. Experimental evidence of potential adverse health effects from industrial food additives alone is not sufficient to prompt regulatory action to reassess the public health safety of UPF additives in relation to disease risk. At minimum, the US Food and Drug Administration (FDA) would need dietary exposure estimates based on repeated intake of additive-containing UPFs and level of additive content along with longitudinal health data, to evaluate whether UPF additive exposure is associated with incident disease and therefore a serious safety issue.

Consequentially, this issue cannot be accurately addressed in the U.S. at this moment because no nationally representative prospective survey captures repeated dietary intake measures alongside participant health profiles across gender and age groups. In addition, most Generally Recognized as Safe (GRAS) food additives commonly used in UPFs lack specified regulatory limits of use and are instead used at the discretion of U.S. manufacturers under Good Manufacturing Practices (GMP) guidelines [6]. In contrast, dietary intake estimates of UPF additive exposure and association with disease risk have been generated using the French NutriNet-Santé prospective cohort (*n* = 108,723; 2009–2023), which integrates multiple composition databases, direct laboratory analyses of food matrices, and relies on a European regulatory system with defined additive-use limits and approved technical functions [7,8,9]. Using the NutriNet-Santé extensive data on additive intake, French researchers have shown associations between specific additive classes (e.g., preservatives) and additive groupings in UPF food categories and multiple chronic diseases [10,11,12,13].

In the U.S., evidence on UPF-related disease risk comes primarily from prospective cohort studies linking intake of UPF food categories, without additive-level data, to incident disease outcomes, notably type 2 diabetes (T2D) [14,15,16]. Several prospective studies have found significant associations between UPF-sweetened beverage intake and incident T2D [14,15]. However, efforts to identify ingredients with health concerns within these UPF categories, such as added sugar, salt, and unhealthy fats, explained less than 30% of the observed disease risk [14]. Moreover, limited information is available on the additive composition of UPF categories, especially GRAS-approved additives with evidence of potential harm. In the U.S., public concern about food additives has largely centred on preservatives, even when adverse health effects have not been established for additives in this functional class. Cautious consumer attitudes about preservatives are reflected in often seen package claims such as “Contains No Preservatives”. Therefore, this cross-sectional study aims to identify additives with existing evidence of harm in artificially sweetened and sugar-sweetened beverages, a UPF category associated with incident T2D and widely consumed across age, sex and socioeconomic status. Using product label information, the study focuses on preservatives, artificial sweeteners, and added sugars by identifying the presence of benzoates, sorbates, and phosphate preservatives, selected artificial sweeteners and the co-occurrence of these additives in sweetened beverages sold by food and beverage manufacturers in 2020. The focus on frequency of occurrence of these three preservatives is based on experimental evidence of potential harm from benzoates and sorbates [17,18] and prior work linking evidence of excessive phosphorus intake from additives to adverse health effects [19], suggesting plausible mechanisms through which these additives may contribute to disease risk. Phosphoric acid and its associated salts are listed by FDA as approved antimicrobials, or preservatives, and higher phosphorus intake from UPF consumption may increase oxidative stress and inflammation, thereby disrupting metabolic processes [19,20]. Prior studies have associated artificially sweetened and sugar-sweetened UPF beverages with T2D risk [21,22,23] and additive mixtures in UPFs with incident diabetes [13]. By documenting how frequently additives with emerging health concerns are used, and in which combinations, within a UPF category linked to incident chronic disease, this study provides information relevant to U.S. policy discussions and future research on potential mechanisms underlying chronic disease risk [24,25].

## 2. Materials and Methods

### 2.1. Data Source

Sales revenue data for the top 25 U.S. food and beverage manufacturers for 2020 were sourced from Euromonitor Passport. A comprehensive list of brands owned by the 25 manufacturers was developed using global and U.S.-based company websites. Ingredient data for all products from these 25 manufacturers were then sourced from Label Insight (a NielsenIQ company, Chicago, IL, USA) in May 2021. The Label Insight database contains information for >400,000 barcoded food and beverages, representing >80% of the U.S. market. For all beverage products, information on brand name, product description, ingredient list, total sugar content (g/100 mL) and product category were extracted from the dataset. Each beverage product was assigned to 1 of 10 Euromonitor Passport categories (and 23 subcategories) to facilitate sales-weighting of results. Products that were missing an ingredient list or total sugar content were excluded from analysis. As this analysis focused on sweetened beverages only, products that did not contain added sugar or non-nutritive sweetener (NNS) were also excluded.

### 2.2. Identification of Sorbates, Benzoates, Phosphate Additives, NNS and Added Sugar

The Food and Drug Administration (FDA) Substances Added to Food inventory, previously known as Everything Added to Foods in the United States, was used to compile the taxonomy used in this study. Table S1 outlines the search terms used to identify benzoates, sorbates, phosphates, NNS and added sugar for this study. For NNS, additives listed as having an NNS function under the Substances Added to Foods inventory or that appear in the FDA’s list of approved high-intensity sweeteners were included.

### 2.3. Data Analysis

Data were analyzed using Stata V18 (Stata Corp, College Station, TX, USA). The total number of products in each category/subcategory was calculated, as was the total revenue (in USD $million) of the included categories/subcategories derived from Euromonitor Passport. The number and proportion of products containing sorbates, benzoates, phosphates, NNS and added sugar ingredients were calculated by beverage category/subcategory. Results were weighted by category sales to generate sales-weighted proportions of products containing each ingredient type to better understand population purchases of sorbates, benzoates, phosphates, NNS and added sugar by their exposure and availability. Sales-weighted proportions were calculated based on each category or subcategory’s sales relative to the total combined sales of all categories, with 2020 sales values obtained from Euromonitor Passport. This approach was taken to apply a weighting that is most relevant for health impact (assuming sales are correlated with consumption). This study did not involve human subject research and thus did not require Institutional Review Board approval.

## 3. Results

Out of 4755 beverage products extracted, 1369 (29%) were removed as they did not contain a sweetening agent or were missing ingredient data, leaving *n* = 3386 products for analysis.

### 3.1. Presence of Preservative Additives

Across all beverages (*n* = 3386), phosphates were the most prevalent preservative additive, identified in 1446 products (42.7%), followed by benzoates (976 products; 28.8%) and sorbates (628 products; 18.5%) (Table 1). Sales-weighted estimates indicated that phosphates were present in approximately 54.0% of beverage sales, while benzoate- and sorbate-containing products were present in 39.0% and 22.3%, respectively. No beverage products contained phosphates, benzoates, or sorbates in isolation.

Preservative use varied by beverage category. *Carbonated beverages* exhibited some of the highest preservative prevalence overall (80% contained benzoates, 16% contained sorbates and 30% contained phosphates). Moreover, *Carbonated beverages* contributed the highest proportion of sales of products containing benzoates (74%) and sorbates (39%) and a high proportion of sales of products containing phosphates (30%). Among *Regular cola carbonates*, 98.0% contained phosphates, reflecting near-universal use of phosphoric acid-based ingredients. In *Low-calorie cola carbonates*, phosphate use was universal (100%), and benzoates (65.9%) and sorbates (40.9%) were substantially more common than in regular colas, but always in combination with phosphates. *Lemonade/lime carbonates* showed high benzoate prevalence (78.2%) and lower sorbate use (10.9%), while *Other non-cola carbonates* contained benzoates in 94.8% of products. Most carbonated subcategories frequently included two or more preservative additives concurrently.

*Energy drinks* demonstrated one of the highest preservative burdens across all categories, with phosphates present in 76.5%, benzoates in 38.3%, and sorbates in 35.6% of products. *Energy drinks* also contributed the largest proportion of sales of products containing phosphates (36%) and the second highest proportion of sales of benzoates (14%) and sorbates (22%). In contrast, ready-to-drink (RTD) coffees and hot drinks rarely contained benzoates or sorbates, although *Hot drinks* had a high use of phosphates (77%). *RTD tea* had a high use of phosphates (38%) and sorbates (37%) but a low use of benzoates (3%). *Dairy* products showed the same trend, with high use of phosphate additives (63%), but low use of benzoates (1%) and sorbates (5%). Despite *Dairy* products having a high use of phosphates (63%), their contribution to sales of products containing phosphates was low (5%). In the *Dairy* category, sorbates were exclusively used in *Drinking yoghurts* (93%), and most products with phosphates in this category were *Coffee whiteners* (79%) and *Other milk alternatives* (74%).

### 3.2. Presence of Sweeteners

Added sugars were prevalent, present in *n* = 2815 products (83.1%). When weighted by sales, added sugar-containing products contributed approximately 75% of total beverage sales, indicating widespread consumer exposure. NNS were present in *n* = 1449 products (43%), with a similar sales-weighted prevalence of 46%. Hybrid sweetening strategies were also common; products containing both added sugar and NNS represented 26% of products and were more common than products containing NNS without added sugar (17%).

Sweetener use varied by category. Although *RTD Coffee*, *Hot Drinks* and *Dairy beverages* had the highest proportion of products containing added sugar (100%, 99% and 99%, respectively), *Carbonated beverages* contributed the largest proportion of sales of products containing added sugar (45%). Interestingly, *Carbonated beverages* had a relatively low proportion of products containing NNS (29%) yet contributed the largest proportion of sales of products containing NNS (31%). *Energy drinks* had the highest proportion of products containing NNS of all beverage categories (84%) and added sugar was present in 56.4% of products. *Energy drinks* also contributed a large proportion of sales of products containing NNS overall (29%) and 40% of products contained both added sugar and NNS in combination. *Sports drinks* were predominantly sweetened with added sugars (73.2%), with NNS used in 3% of products. *Sports drinks* containing NNS in combination with added sugars were rare (1%), indicating that NNS and added sugar were generally used in isolation as a sweetening agent. Within *Bottled waters*, NNS was more commonly used as a sweetening agent (64%) compared to added sugar (47%), with 12% of products containing both NNS and added sugar in combination. *Concentrates* and *RTD Tea* were the categories with the highest combined use of NNS and added sugar (54% and 42%, respectively).

### 3.3. Co-Occurrence of Sweeteners and Preservative Additives

Sweeteners and preservative additives showed common co-occurrence across the beverage supply. Among all sweetened beverages, over 70% of products contained at least one preservative additive, and a substantial proportion contained two or more preservatives concurrently (Table S2). Across beverage categories, 30–45% of added sugar-containing products also contained phosphates, 25–40% contained benzoates, with sorbates present in a smaller but still notable proportion (15–25%, depending on category). Multi-preservative formulations combining benzoates and sorbates were common. Products containing NNS exhibited higher additive clustering compared to products containing added sugar. Across categories, more than half of NNS-containing products also included phosphates, and approximately one-third to one-half also contained benzoates. Products containing both added sugar and NNS were consistently more likely to include multiple preservative additives than products sweetened with added sugar alone, with three- and four-additive combinations representing the majority of hybrid-sweetened products. Among preservative additives, phosphates emerged as a dominant co-exposure, appearing in 40–75% of products across major beverage categories and frequently co-occurring with both added sugar and NNS.

Carbonated beverages exhibited some of the highest levels of additive complexity. In *Regular cola products*, nearly all (>95%) combined added sugars with phosphates, and approximately 20–25% also included benzoates or sorbates in addition to phosphates. In *Low-calorie cola products*, formulations containing added sugar and NNS plus one or more preservatives accounted for over half of products, while NNS-only formulations remained rare (<5%). *Non-cola carbonated beverages* showed particularly strong reliance on added sugar + benzoate combinations, accounting for over 40% of products, with an additional 15–20% also including phosphates or sorbates.

*Juices* demonstrated diverse but additive-dense profiles. Approximately 80% of products combined added sugars with at least one preservative, most commonly benzoates or phosphates. Hybrid-sweetened products (added sugar + NNS) accounted for 15–20% of formulations and were more likely than added sugar-only products to include two or more preservative additives.

*Energy drinks* showed some of the most pronounced additive stacking. Over 70% of products contained phosphates, and approximately 40% contained benzoates, most often in combination with either added sugar alone or added sugar + NNS. Products containing three or more additive classes accounted for the majority of energy drinks, with NNS-containing products disproportionately represented among the most additive-dense formulations.

*Sports drinks* were characterized by frequent co-use of added sugar and NNS, with over 40% of products containing both sweeteners. NNS-only formulations accounted for <3% of products. Phosphates were present in over 90% of sports drinks, and approximately 35–40% also included sorbates or benzoates, resulting in frequent three- and four-additive combinations.

Within *Bottled waters*, additive complexity varied by subcategory. *Functional bottled waters* commonly combined NNS with phosphates, accounting for approximately 25–30% of products, with a smaller subset also including benzoates or sorbates. *Carbonated bottled waters* more frequently combined added sugar with phosphates, though multi-preservative formulations were less common than in *Carbonated soft drinks*.

*RTD teas* frequently combined added sugars with benzoates and/or sorbates, accounting for over 50% of products, with 10–15% also including NNS. In contrast, *RTD coffees* were more often characterized by added sugar-based formulations with fewer preservative additives, though multi-additive combinations were still present in approximately 30–40% of products.

**Figure 1:**
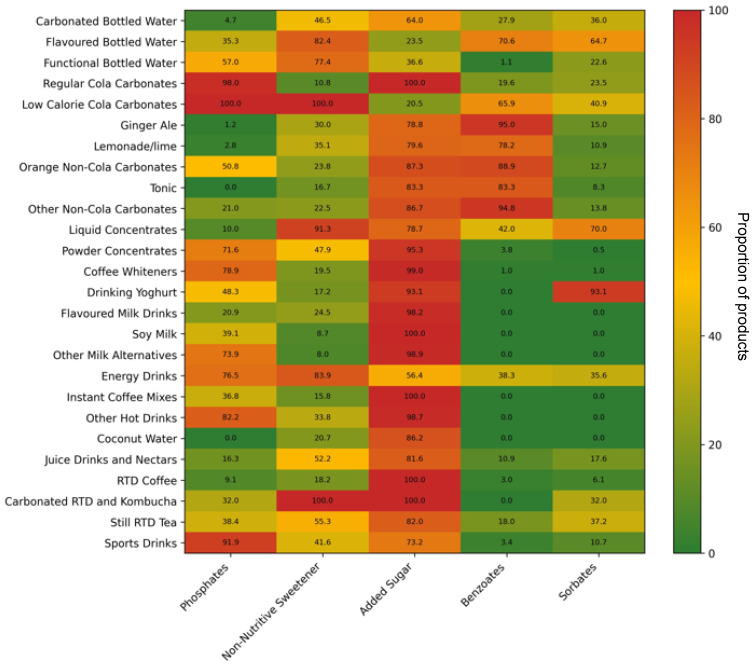
Heat map showing proportion of US sweetened beverage products containing non-nutritive sweetener, added sugar, phosphates, benzoates and sorbates.

Figure 1 allows visualization of the percentage of products in each beverage category that contained each additive. The heatmap uses different colours to represent increasing percentages as shown in the key.

## 4. Discussion

This study characterized the occurrence and co-occurrence of selected additive classes within sweetened beverages sold in the U.S. Multiple additive types frequently appeared together within beverage formulations, with combinations of sweeteners, preservatives and phosphate additives more common than simpler formulations. These findings highlight the complexity of beverage formulations and provide a descriptive overview of additive co-occurrence patterns within the U.S. beverage supply. Additive mixes specific to food groups have previously been identified using French dietary survey data [7,10,11,12,13].

Sweetened beverages are widely researched in relation to metabolic disease risk, yet research has largely focused on isolated nutrients or ingredients, particularly added sugars or NNS, rather than the broader formulation context in which these compounds are consumed. One notable finding from this study was that products containing both added sugar and NNS were more prevalent than products containing NNS in isolation, suggesting that hybrid sweetening strategies are common in the sweetened beverage supply. This observation suggests that future dietary and epidemiologic studies may benefit from considering sweetener combinations rather than treating sugar-sweetened and NNS sweetened beverages as mutually exclusive. Many products may expose consumers simultaneously to added sugar and NNS combining with other ingredients to form a “cocktail effect” that is well-recognized and hypothesized to impact critical aspects of metabolic disruption, although the specific biological outcomes of such additive mixing remain speculative and unclear [13,25].

An important observation in the current study is that beverages containing NNS tended to be among the most additive-dense formulations identified in the dataset. Products containing NNS were more likely to contain preservatives such as benzoates and sorbates, as well as phosphate additives, compared with beverages sweetened with added sugar alone. Many NNS-containing beverages fell within formulations containing three or four of these additives simultaneously. This is an important consideration given that beverages marketed as “diet,” “zero sugar,” or “reduced sugar” are often perceived by consumers as healthier alternatives. However, while these products may contain less sugar (or in some cases no added sugar), they instead contain more complex additive profiles. This raises important questions regarding whether reformulation strategies focused primarily on sugar reduction may inadvertently increase exposure to other additives with their own uncertain long-term health implications. Differences between products sweetened with added sugar and those sweetened with a combination of added sugar and NNS also suggest that additive ingredients vary depending on the sweetening approach used. While sugar-sweetened beverages frequently contained preservative additives, products containing both added sugars and NNS were even more likely to include multiple preservative types concurrently. Future research should therefore move beyond isolated sweetener exposures and consider the broader formulation characteristics in addition to sweetener composition.

Results also demonstrated that phosphates frequently co-occurred with both added sugars and NNS, especially in categories such as carbonated soft drinks, energy drinks, and sports drinks, raising concerns about a potential role for repeated co-occurrence and cumulative effects of phosphate additives in these products. Although phosphate additives have historically received less attention in nutrition epidemiology compared to added sugar or NNS, their consistent presence within highly formulated beverages warrants further investigation. Future research merging additive concentrations and dietary intake data may help clarify the true extent of phosphate exposure from beverages. We believe the strongest clinical evidence of additive harm is with a cumulative effect of phosphate additive intake where evidence of hormonal dysregulation of phosphorus homeostasis adversely impacts kidney function and increases cardiovascular calcification [20,26,27]. Epidemiologic studies have shown that these beverage categories are associated with increased risk of incident T2D [14,15]. Furthermore, 56% of over 40,000 UPF products sold in the U.S. in 2020 contained phosphate additives with various technical functions, suggesting significant potential for a cumulative effect on excess phosphate intake [28]. The widespread use of phosphate additives in UPFs has been suggested as one of many potential mechanisms linking UPF consumption to chronic disease risk [29].

On the other hand, our hypothesis linking individual and co-occurring preservative additives, NNS, and added sugars is based on the concept of a shared mechanism that contributes to chronic disease risk: promotion of advanced glycation end-product (AGE) formation in the beverage matrix and possibly in the gastrointestinal tract after absorption. AGE formation is known to promote oxidative stress and inflammation, which are underlying processes in many chronic diseases [30,31]. Although speculative, this hypothesis is supported by experimental observations. Emami and colleagues recently reported that in vitro binding of sodium benzoate and potassium sorbate to bovine serum albumin significantly increased AGE synthesis [32], and earlier in vitro work by Rizzi [33] showed that phosphate ions stimulated the Maillard reaction and increased AGE formation. Added sugars may also accelerate protein glycation, especially high-fructose corn syrup (HFCS), the most common added sugar in UPFs in the U.S. By increasing exposure to glucose and fructose, HFCS-containing soft drinks have been shown to increase AGE production both in the intestinal lumen [34] and endogenously [35]. Evidence for NNS effects on AGE formation remains limited and weak. A meta-analysis in diabetic patients found that, across three trials comparing NNS with sugar, the NNS group had a non-clinically significant 0.4% higher glycosylated hemoglobin (HbA1c) [36]. A more recent meta-analysis comparing stevia with sugar found no change in HbA1c in either group [37]. Deo et al. [38] examined NNS-related AGE formation in vitro using exogenous and endogenous models. In the exogenous model, xylitol and sorbitol produced higher levels of specific AGEs than sucralose; in the endogenous model (37 °C), sucralose produced higher levels of different AGEs than sugar alcohols. Evidence on soft-drink additive mixtures that promote AGE formation is also limited. However, Taghavi et al. [39] showed that in vitro mixtures of potassium sorbate and glucose activated AGE synthesis from human serum albumin, supporting the biological plausibility of endogenous AGE formation from known additive-sugar mixtures [13]. Understanding how combinations of sweeteners and preservatives interact biologically may be important for clarifying pathways linking ultra-processed beverage consumption with metabolic disease risk. A plausible mechanistic link between these additives and increased formation of AGEs merits further studies [25].

These findings have several implications. First, current dietary surveillance systems and epidemiologic studies underestimate the complexity of additive exposures arising from ultra-processed beverages. Second, regulatory and reformulation strategies that focus solely on reducing sugar content may fail to account for compensatory increased use of other chemical additives. Finally, these findings highlight the need for future mechanistic and longitudinal studies examining additive mixtures and cumulative exposures rather than single additives or ingredients in isolation.

This study has several strengths, including the use of a large, nationally representative food database and a systematic approach to ingredient disaggregation and classification. The analysis extends beyond conventional nutrient-focused approaches by examining co-occurrence patterns with sweeteners and preservative additives. However, product ingredient lists do not provide quantitative information, preventing estimation of additive levels per serving. The analysis is cross-sectional and limited to products available at the time of data collection in 2020. As new products are continuously introduced to the market, and products are discontinued, it is likely that there are new products that were not captured in our analysis and that some products have been discontinued.

## 5. Conclusions

This study examined the occurrence and co-occurrence of selected preservatives, NNS, added sugar and phosphate additives in ultra-processed sweetened beverages sold in the U.S. Multiple additive types frequently appeared together, with products containing NNS often exhibiting the most complex additive formulations. These findings highlight an important but understudied characteristic of UPFs; their complexity extends beyond nutrient composition to include combinations of multiple additives consumed simultaneously. While this study did not assess additive concentrations, dietary intake or health outcomes, the widespread occurrence of UPF ingredients supports the need for future research examining additive mixtures as a potentially relevant dimension of UPF consumption.

## Figures and Tables

**Table 1:** Proportion of US sweetened beverages containing non-nutritive sweetener, added sugar, phosphates, sorbates and benzoates.

Category/Subcategory	Contains NNS Only		Contains Added Sugar Only		Contains Phosphates		Contains NNS	
	N (%)	% Sales	N (%)	% Sales	N (%)	% Sales	N (%)	% Sales
Bottled Water (*n* = 196)	42 (21%)	31%	44 (22%)	7%	63 (32%)	3%	126 (64%)	12%
Carbonated Bottled Water (*n* = 86)	12 (14%)	3%	36 (42%)	4%	4 (5%)	0%	40 (47%)	1%
Flavoured Bottled Water (*n* = 17)	4 (24%)	12%	1 (6%)	1%	6 (35%)	1%	14 (82%)	5%
Functional Bottled Water (*n* = 93)	26 (28%)	16%	7 (8%)	2%	53 (57%)	2%	72 (77%)	6%
Carbonates (*n* = 859)	9 (1%)	5%	38 (4%)	8%	256 (30%)	23%	248 (29%)	31%
Regular Cola Carbonates (*n* = 102)	0 (0%)	0%	2 (2%)	2%	100 (98%)	9%	11 (11%)	3%
Low-Calorie Cola Carbonates (*n* = 44)	0 (0%)	0%	0 (0%)	0%	44 (100%)	10%	44 (100%)	15%
Ginger Ale (*n* = 80)	2 (3%)	1%	1 (1%)	0%	1 (1%)	0%	24 (30%)	1%
Lemonade/lime (*n* = 211)	2 (1%)	1%	24 (11%)	3%	6 (3%)	0%	74 (35%)	3%
Orange Non-Cola Carbonates (*n* = 63)	0 (0%)	0%	4 (6%)	1%	32 (51%)	1%	15 (24%)	1%
Tonic (*n* = 12)	0 (0%)	0%	1 (8%)	0%	0 (0%)	0%	2 (17%)	0%
Other Non-Cola Carbonates (*n* = 347)	5 (1%)	4%	6 (2%)	2%	73 (21%)	3%	78 (22%)	8%
Concentrates (*n* = 361)	6 (2%)	0%	22 (6%)	1%	166 (46%)	2%	238 (66%)	2%
Liquid Concentrates (*n* = 150)	2 (1%)	0%	6 (4%)	0%	15 (10%)	0%	137 (91%)	1%
Powder Concentrates (*n* = 211)	4 (2%)	0%	16 (8%)	1%	151 (72%)	2%	101 (48%)	1%
Dairy (*n* = 553)	1 (0%)	0%	171 (31%)	22%	350 (63%)	5%	100 (18%)	3%
Coffee Whiteners (*n* = 303)	0 (0%)	0%	61 (20%)	1%	239 (79%)	1%	59 (19%)	0%
Drinking Yoghurt (*n* = 29)	0 (0%)	0%	0 (0%)	0%	14 (48%)	0%	5 (17%)	0%
Flavoured Milk Drinks (*n* = 110)	1 (1%)	0%	76 (69%)	15%	23 (21%)	3%	27 (25%)	2%
Soy Milk (*n* = 23)	0 (0%)	0%	12 (52%)	1%	9 (39%)	0%	2 (9%)	0%
Other Milk Alternatives (*n* = 88)	0 (0%)	0%	22 (25%)	4%	65 (74%)	1%	7 (8%)	0%
Energy Drinks (*n* = 149)	17 (11%)	27%	9 (6%)	6%	114 (77%)	36%	125 (84%)	26%
Hot Drinks (*n* = 176)	0 (0%)	0%	33 (19%)	1%	136 (77%)	1%	56 (32%)	0%
Instant Coffee Mixes (*n* = 19)	0 (0%)	0%	10 (53%)	1%	7 (37%)	0%	3 (16%)	0%
Other Hot Drinks (*n* = 157)	0 (0%)	0%	23 (15%)	1%	129 (82%)	1%	53 (34%)	0%
Juice (*n* = 552)	90 (16%)	18%	226 (41%)	18%	85 (15%)	4%	279 (51%)	7%
Coconut Water (*n* = 29)	4 (14%)	0%	23 (79%)	1%	0 (0%)	0%	6 (21%)	0%
Juice Drinks and Nectars (*n* = 523)	86 (16%)	18%	203 (39%)	17%	85 (16%)	4%	273 (52%)	7%
RTD Coffee (*n* = 33)	0 (0%)	0%	26 (79%)	20%	3 (9%)	2%	6 (18%)	1%
RTD Tea (*n* = 358)	40 (11%)	13%	117 (33%)	16%	136 (38%)	13%	209 (58%)	9%
Carbonated RTD and Kombucha (*n* = 25)	0 (0%)	0%	0 (0%)	0%	8 (32%)	2%	25 (100%)	2%
Still RTD Tea (*n* = 333)	40 (12%)	13%	117 (35%)	16%	128 (38%)	12%	184 (55%)	8%
Sports Drinks (*n* = 149)	4 (3%)	4%	2 (1%)	1%	137 (92%)	9%	62 (42%)	8%
Total (*n* = 3386)	209 (6%)	100%	688 (20%)	100%	1446 (43%)	100%	1449 (43%)	100%
Sales-weighted proportion	6%		14%		54%		46%	
	**Contains added sugar**		**Contains benzoates**		**Contains sorbates**			
Bottled Water (*n* = 196)	93 (47%)	4%	37 (19%)	6%	63 (32%)	14%		
Carbonated Bottled Water (*n* = 86)	55 (64%)	1%	24 (28%)	1%	31 (36%)	2%		
Flavoured Bottled Water (*n* = 17)	4 (24%)	1%	12 (71%)	5%	11 (65%)	9%		
Functional Bottled Water (*n* = 93)	34 (37%)	2%	1 (1%)	0%	21 (23%)	3%		
Carbonates (*n* = 859)	708 (82%)	45%	685 (80%)	74%	134 (16%)	39%		
Regular Cola Carbonates (*n* = 102)	102 (100%)	15%	20 (20%)	6%	24 (24%)	12%		
Low-Calorie Cola Carbonates (*n* = 44)	9 (20%)	2%	29 (66%)	11%	18 (41%)	12%		
Ginger Ale (*n* = 80)	63 (79%)	1%	76 (95%)	3%	12 (15%)	1%		
Lemonade/lime (*n* = 211)	168 (80%)	4%	165 (78%)	8%	23 (11%)	2%		
Orange Non-Cola Carbonates (*n* = 63)	55 (87%)	3%	56 (89%)	5%	8 (13%)	1%		
Tonic (*n* = 12)	10 (83%)	1%	10 (83%)	1%	1 (8%)	0%		
Other Non-Cola Carbonates (*n* = 347)	301 (87%)	19%	329 (95%)	39%	48 (14%)	10%		
Concentrates (*n* = 361)	319 (88%)	2%	71 (20%)	1%	106 (29%)	1%		
Liquid Concentrates (*n* = 150)	118 (79%)	0%	63 (42%)	0%	105 (70%)	1%		
Powder Concentrates (*n* = 211)	201 (95%)	2%	8 (4%)	0%	1 (0%)	0%		
Dairy (*n* = 553)	545 (99%)	9%	3 (1%)	0%	30 (5%)	1%		
Coffee Whiteners (*n* = 303)	300 (99%)	1%	3 (1%)	0%	3 (1%)	0%		
Drinking Yoghurt (*n* = 29)	27 (93%)	0%	0 (0%)	0%	27 (93%)	1%		
Flavoured Milk Drinks (*n* = 110)	108 (98%)	4%	0 (0%)	0%	0 (0%)	0%		
Soy Milk (*n* = 23)	23 (100%)	0%	0 (0%)	0%	0 (0%)	0%		
Other Milk Alternatives (*n* = 88)	87 (99%)	3%	0 (0%)	0%	0 (0%)	0%		
Energy Drinks (*n* = 149)	84 (56%)	11%	57 (38%)	14%	53 (36%)	22%		
Hot Drinks (*n* = 176)	174 (99%)	1%	0 (0%)	0%	0 (0%)	0%		
Instant Coffee Mixes (*n* = 19)	19 (100%)	0%	0 (0%)	0%	0 (0%)	0%		
Other Hot Drinks (*n* = 157)	155 (99%)	1%	0 (0%)	0%	0 (0%)	0%		
Juice (*n* = 552)	452 (82%)	7%	57 (10%)	2%	92 (17%)	5%		
Coconut Water (*n* = 29)	25 (86%)	0%	0 (0%)	0%	0 (0%)	0%		
Juice Drinks and Nectars (*n* = 523)	427 (82%)	7%	57 (11%)	2%	92 (18%)	5%		
RTD Coffee (*n* = 33)	33 (100%)	5%	1 (3%)	0%	2 (6%)	1%		
RTD Tea (*n* = 358)	298 (83%)	8%	60 (17%)	3%	132 (37%)	12%		
Carbonated RTD and Kombucha (*n* = 25)	25 (100%)	1%	0 (0%)	0%	8 (32%)	1%		
Still RTD Tea (*n* = 333)	273 (82%)	7%	60 (18%)	3%	124 (37%)	11%		
Sports Drinks (*n* = 149)	109 (73%)	9%	5 (3%)	1%	16 (11%)	4%		
Total (*n* = 3386)	2815 (83%)	100%	976 (29%)	100%	628 (19%)	100%		
Sales-weighted proportion	75%		39%		22%			

## Data Availability

Data cannot be shared due to the proprietary nature of the Label Insight data.

## Supplementary Materials

The following supporting information can be downloaded at https://www.mdpi.com/article/10.3390/nu18132176/s1. Table S1: Search terms for non-nutritive sweeteners, added sugars, phosphates, benzoates and sorbates. Table S2: Number and proportion of US sweetened beverage products containing each ingredient combination.
