# Supplementary material for: Additives with Emerging Health Concerns in Ultra-Processed Sweetened Beverages Sold in the United States: Preservatives, Artificial Sweeteners, and Added Sugars
Source: Nutrients. 2026 Jul 4;18(13):2176. doi: 10.3390/nu18132176 (PMC13363947; doi:10.3390/nu18132176)
Supplement: Supplementary file 1 [file nutrients-18-02176-s001.zip › nutrients-4368697-supplementary.pdf]

Table S1: Search terms for non-nutritive sweetener, added sugar, phosphates, benzoates and sorbates

Ingredient	Search terms
Non-nutritive sweetener	Acesulfame k; aspartame; erythritol; isomaltulose; monk fruit; monk fruit extract; neotame; oligofructose; stevia; stevia extract; stevia leaf; stevia leaf extract; steviol glycosides; sucralose; erythritol; isomaltulose; mannitol; sorbitol
Benzoates	Benzoate
Sorbates	Sorbate
Phosphates	Phosphoric acid salts (orthophosphates); pyrophosphates; polyphosphates; lecithins; modified food starches; glycerol phosphates; guanosine phosphates; inositol phosphates; Riboflavin-5'-phosphate
Added sugar	Agave syrup; barley extract; cane sugar; caramel syrup; caramelized sugar; coconut sugar; corn syrup; corn syrup solids; crystalline fructose; d-ribose; dried cane syrup; dried corn syrup; dried molasses; evaporated cane sugar; fructooligosaccharides; fructose; fruit sugar; glucose; glucose syrup; high fructose corn syrup; high fructose corn syrup and/or sucrose; honey; invert sugar; liquid sugar; maple extract; maple syrup; molasses; oat extract; pure cane sugar; ribose; sucrose; sugar; tapioca syrup

<u>Table S2:</u> Number and proportion of US sweetened beverage products containing each ingredient combination.

Category	AS+ NNS	AS+ benzoates	AS+ sorbates	AS+ phosphates	NNS+ benzoates	NNS+ phosphates	NNS+ sorbates	AS+NNS+ benzoates
**Bottled Water (n=196)**	6 (3%)	0 (0%)	8 (4%)	14 (7%)	3 (2%)	14 (7%)	0 (0%)	3 (2%)
Carbonated Bottled Water (n=86)	0 (0%)	0 (0%)	8 (9%)	1 (1%)	3 (3%)	0 (0%)	0 (0%)	3 (3%)
Flavoured Bottled Water (n=17)	0 (0%)	0 (0%)	0 (0%)	0 (0%)	0 (0%)	0 (0%)	0 (0%)	0 (0%)
Functional Bottled Water (n=93)	6 (6%)	0 (0%)	0 (0%)	13 (14%)	0 (0%)	14 (15%)	0 (0%)	0 (0%)
**Carbonates (n=859)**	12 (1%)	354 (41%)	8 (1%)	59 (7%)	77 (9%)	0 (0%)	0 (0%)	36 (4%)
Regular Cola Carbonates (n=102)	0 (0%)	0 (0%)	0 (0%)	58 (57%)	0 (0%)	0 (0%)	0 (0%)	0 (0%)
Low Calorie Cola Carbonates (n=44)	0 (0%)	0 (0%)	0 (0%)	0 (0%)	0 (0%)	0 (0%)	0 (0%)	0 (0%)
Ginger Ale (n=80)	0 (0%)	45 (56%)	0 (0%)	0 (0%)	14 (18%)	0 (0%)	0 (0%)	6 (8%)
Lemonade/lime (n=211)	8 (4%)	101 (48%)	6 (3%)	0 (0%)	37 (18%)	0 (0%)	0 (0%)	13 (6%)
Orange Non-Cola Carbonates (n=63)	1 (2%)	18 (29%)	1 (2%)	0 (0%)	4 (6%)	0 (0%)	0 (0%)	1 (2%)
Tonic (n=12)	0 (0%)	8 (67%)	1 (8%)	0 (0%)	2 (17%)	0 (0%)	0 (0%)	0 (0%)
Other Non-Cola Carbonates (n=347)	3 (1%)	182 (52%)	0 (0%)	1 (0%)	20 (6%)	0 (0%)	0 (0%)	16 (5%)
**Concentrates (n=361)**	64 (18%)	3 (1%)	0 (0%)	96 (27%)	0 (0%)	4 (1%)	16 (4%)	4 (1%)
Liquid Concentrates (n=150)	28 (19%)	2 (1%)	0 (0%)	4 (3%)	0 (0%)	1 (1%)	16 (11%)	2 (1%)
Powder Concentrates (n=211)	36 (17%)	1 (0%)	0 (0%)	92 (44%)	0 (0%)	3 (1%)	0 (0%)	2 (1%)
**Dairy (n=553)**	13 (2%)	0 (0%)	11 (2%)	260 (47%)	1 (1%)	2 (0%)	2 (0%)	0 (0%)
Coffee Whiteners (n=303)	0 (0%)	0 (0%)	0 (0%)	183 (60%)	0 (0%)	0 (0%)	0 (0%)	0 (0%)
Drinking Yoghurt (n=29)	0 (0%)	0 (0%)	11 (38%)	2 (7%)	0 (0%)	0 (0%)	2 (7%)	0 (0%)
Flavoured Milk Drinks (n=110)	10 (9%)	0 (0%)	0 (0%)	7 (6%)	0 (0%)	1 (1%)	0 (0%)	0 (0%)
Soy Milk (n=23)	2 (9%)	0 (0%)	0 (0%)	9 (39%)	0 (0%)	0 (0%)	0 (0%)	0 (0%)
Other Milk Alternatives (n=88)	1 (1%)	0 (0%)	0 (0%)	59 (67%)	0 (0%)	1 (1%)	0 (0%)	0 (0%)
**Energy Drinks (n=149)**	4 (3%)	1 (1%)	0 (0%)	6 (4%)	1 (1%)	22 (15%)	0 (0%)	1 (1%)
**Hot Drinks (n=176)**	7 (4%)	0 (0%)	0 (0%)	87 (49%)	0 (0%)	2 (1%)	0 (0%)	0 (0%)
Instant Coffee Mixes (n=19)	2 (11%)	0 (0%)	0 (0%)	6 (32%)	0 (0%)	0 (0%)	0 (0%)	0 (0%)
Other Hot Drinks (n=157)	5 (3%)	0 (0%)	0 (0%)	81 (52%)	0 (0%)	2 (1%)	0 (0%)	0 (0%)

**Juice (n=552)**	120 (22%)	3 (1%)	6 (1%)	5 (1%)	0 (0%)	8 (1%)	0 (0%)	1 (0%)
Coconut Water (n=29)	2 (7%)	0 (0%)	0 (0%)	0 (0%)	0 (0%)	0 (0%)	0 (0%)	0 (0%)
Juice Drinks and Nectars (n=523)	118 (23%)	3 (1%)	6 (1%)	5 (1%)	0 (0%)	8 (2%)	0 (0%)	1 (0%)
**RTD Coffee (n=33)**	3 (9%)	0 (0%)	0 (0%)	1 (3%)	0 (0%)	0 (0%)	0 (0%)	0 (0%)
**RTD Tea (n=358)**	45 (13%)	0 (0%)	2 (1%)	19 (5%)	0 (0%)	5 (1%)	2 (1%)	0 (0%)
Carbonated RTD and Kombucha (n=25)	17 (68%)	0 (0%)	0 (0%)	0 (0%)	0 (0%)	0 (0%)	0 (0%)	0 (0%)
Still RTD Tea (n=333)	28 (8%)	0 (0%)	2 (1%)	19 (6%)	0 (0%)	5 (2%)	2 (1%)	0 (0%)
**Sports Drinks (n=149)**	1 (1%)	0 (0%)	0 (0%)	85 (57%)	0 (0%)	21 (14%)	0 (0%)	0 (0%)
**Total (n=3386)**	**275 (8%)**	**361 (11%)**	**35 (1%)**	**632 (19%)**	**81 (2%)**	**78 (2%)**	**20 (1%)**	**45 (1%)**

Table S2 (continued): Number and proportion of US sweetened beverage products containing each ingredient combination.

Category	AS+NNS+ phosphates	AS+NNS +sorbates	AS+benzoates +sorbates	AS+phosphates+ benzoates	AS+phosphates +sorbates	NNS+benzoates +sorbates	NNS+benzoates+ phosphates
**Bottled Water (n=196)**	6 (3%)	2 (1%)	2 (1%)	0 (0%)	0 (0%)	22 (11%)	1 (1%)
Carbonated Bottled Water (n=86)	0 (0%)	2 (2%)	1 (1%)	0 (0%)	0 (0%)	16 (19%)	0 (0%)
Flavoured Bottled Water (n=17)	0 (0%)	0 (0%)	0 (0%)	0 (0%)	0 (0%)	6 (35%)	1 (6%)
Functional Bottled Water (n=93)	6 (6%)	0 (0%)	1 (1%)	0 (0%)	0 (0%)	0 (0%)	0 (0%)
**Carbonates (n=859)**	3 (0%)	10 (1%)	45 (5%)	75 (9%)	18 (2%)	9 (1%)	45 (5%)
Regular Cola Carbonates (n=102)	3 (3%)	0 (0%)	0 (0%)	11 (11%)	17 (17%)	0 (0%)	0 (0%)
Low Calorie Cola Carbonates (n=44)	0 (0%)	0 (0%)	0 (0%)	0 (0%)	0 (0%)	0 (0%)	26 (59%)
Ginger Ale (n=80)	0 (0%)	0 (0%)	9 (11%)	0 (0%)	1 (1%)	1 (1%)	0 (0%)
Lemonade/lime (n=211)	0 (0%)	6 (3%)	1 (0%)	2 (1%)	0 (0%)	3 (1%)	1 (0%)
Orange Non-Cola Carbonates (n=63)	0 (0%)	1 (2%)	1 (2%)	20 (32%)	0 (0%)	0 (0%)	3 (5%)
Tonic (n=12)	0 (0%)	0 (0%)	0 (0%)	0 (0%)	0 (0%)	0 (0%)	0 (0%)
Other Non-Cola Carbonates (n=347)	0 (0%)	3 (1%)	34 (10%)	42 (12%)	0 (0%)	5 (1%)	15 (4%)
**Concentrates (n=361)**	52 (14%)	27 (7%)	1 (0%)	1 (0%)	0 (0%)	9 (2%)	3 (1%)
Liquid Concentrates (n=150)	0 (0%)	27 (18%)	1 (1%)	0 (0%)	0 (0%)	9 (6%)	0 (0%)
Powder Concentrates (n=211)	52 (25%)	0 (0%)	0 (0%)	1 (0%)	0 (0%)	0 (0%)	3 (1%)

**Dairy (n=553)**	76 (14%)	2 (0%)	0 (0%)	0 (0%)	11 (2%)	3 (1%)	0 (0%)
Coffee Whiteners (n=303)	56 (18%)	0 (0%)	0 (0%)	0 (0%)	0 (0%)	3 (1%)	0 (0%)
Drinking Yoghurt (n=29)	0 (0%)	2 (7%)	0 (0%)	0 (0%)	11 (38%)	0 (0%)	0 (0%)
Flavoured Milk Drinks (n=110)	15 (14%)	0 (0%)	0 (0%)	0 (0%)	0 (0%)	0 (0%)	0 (0%)
Soy Milk (n=23)	0 (0%)	0 (0%)	0 (0%)	0 (0%)	0 (0%)	0 (0%)	0 (0%)
Other Milk Alternatives (n=88)	5 (6%)	0 (0%)	0 (0%)	0 (0%)	0 (0%)	0 (0%)	0 (0%)
**Energy Drinks (n=149)**	15 (10%)	1 (1%)	0 (0%)	3 (2%)	0 (0%)	1 (1%)	10 (7%)
**Hot Drinks (n=176)**	47 (27%)	0 (0%)	0 (0%)	0 (0%)	0 (0%)	0 (0%)	0 (0%)
Instant Coffee Mixes (n=19)	1 (5%)	0 (0%)	0 (0%)	0 (0%)	0 (0%)	0 (0%)	0 (0%)
Other Hot Drinks (n=157)	46 (29%)	0 (0%)	0 (0%)	0 (0%)	0 (0%)	0 (0%)	0 (0%)
**Juice (n=552)**	7 (1%)	1 (0%)	11 (2%)	0 (0%)	3 (1%)	1 (0%)	0 (0%)
Coconut Water (n=29)	0 (0%)	0 (0%)	0 (0%)	0 (0%)	0 (0%)	0 (0%)	0 (0%)
Juice Drinks and Nectars (n=523)	7 (1%)	1 (0%)	11 (2%)	0 (0%)	3 (1%)	1 (0%)	0 (0%)
**RTD Coffee (n=33)**	1 (3%)	1 (3%)	0 (0%)	0 (0%)	0 (0%)	0 (0%)	0 (0%)
**RTD Tea (n=358)**	0 (0%)	10 (3%)	2 (1%)	0 (0%)	2 (1%)	3 (1%)	0 (0%)
Carbonated RTD and Kombucha (n=25)	0 (0%)	0 (0%)	0 (0%)	0 (0%)	0 (0%)	0 (0%)	0 (0%)
Still RTD Tea (n=333)	0 (0%)	10 (3%)	2 (1%)	0 (0%)	2 (1%)	3 (1%)	0 (0%)
**Sports Drinks (n=149)**	20 (13%)	0 (0%)	0 (0%)	0 (0%)	0 (0%)	5 (3%)	0 (0%)
**Total (n=3386)**	**227 (7%)**	**54 (2%)**	**61 (2%)**	**79 (2%)**	**34 (1%)**	**53 (2%)**	**59 (2%)**

<u>Table S2 (continued):</u> Number and proportion of US sweetened beverage products containing each ingredient combination.

Category	NNS+sorbates+ phosphates	AS+NNS+ benzoates+ sorbates	AS+NNS+ phosphates+ benzoates	AS+NNS+ phosphates+ sorbates	AS+benzoates+ sorbates+ phosphates	NNS+benzoates+ sorbates+ phosphates	All 5
**Bottled Water (n=196)**	19 (10%)	1 (1%)	0 (0%)	4 (2%)	2 (1%)	2 (1%)	1 (1%)
Carbonated Bottled Water (n=86)	0 (0%)	1 (1%)	0 (0%)	3 (3%)	0 (2%)	0 (0%)	0 (0%)
Flavoured Bottled Water (n=17)	0 (0%)	0 (0%)	0 (0%)	0 (0%)	2 (12%)	2 (12%)	1 (6%)
Functional Bottled Water (n=93)	19 (20%)	0 (0%)	0 (0%)	1 (1%)	0 (0%)	0 (0%)	0 (0%)

**Carbonates (n=859)**	6 (1%)	5 (1%)	17 (2%)	11 (1%)	14 (2%)	5 (1%)	3 (0%)
Regular Cola Carbonates (n=102)	0 (0%)	0 (0%)	4 (4%)	2 (2%)	3 (3%)	0 (0%)	2 (2%)
Low Calorie Cola Carbonates (n=44)	6 (14%)	0 (0%)	0 (0%)	9 (20%)	0 (0%)	3 (7%)	0 (0%)
Ginger Ale (n=80)	0 (0%)	1 (1%)	0 (0%)	0 (0%)	0 (0%)	0 (0%)	0 (0%)
Lemonade/lime (n=211)	0 (0%)	4 (2%)	0 (0%)	0 (0%)	3 (1%)	0 (0%)	0 (0%)
Orange Non-Cola Carbonates (n=63)	0 (0%)	0 (0%)	4 (6%)	0 (0%)	4 (6%)	1 (2%)	0 (0%)
Tonic (n=12)	0 (0%)	0 (0%)	0 (0%)	0 (0%)	0 (0%)	0 (0%)	0 (0%)
Other Non-Cola Carbonates (n=347)	0 (0%)	0 (0%)	9 (3%)	0 (0%)	4 (1%)	1 (0%)	1 (0%)
**Concentrates (n=361)**	1 (0%)	43 (12%)	0 (0%)	2 (1%)	0 (0%)	3 (1%)	4 (1%)
Liquid Concentrates (n=150)	1 (1%)	42 (28%)	0 (0%)	2 (1%)	0 (0%)	3 (2%)	4 (3%)
Powder Concentrates (n=211)	0 (0%)	1 (0%)	0 (0%)	0 (0%)	0 (0%)	0 (0%)	0 (0%)
**Dairy (n=553)**	0 (0%)	0 (0%)	0 (0%)	1 (0%)	0 (0%)	0 (0%)	0 (0%)
Coffee Whiteners (n=303)	0 (0%)	0 (0%)	0 (0%)	0 (0%)	0 (0%)	0 (0%)	0 (0%)
Drinking Yoghurt (n=29)	0 (0%)	0 (0%)	0 (0%)	1 (3%)	0 (0%)	0 (0%)	0 (0%)
Flavoured Milk Drinks (n=110)	0 (0%)	0 (0%)	0 (0%)	0 (0%)	0 (0%)	0 (0%)	0 (0%)
Soy Milk (n=23)	0 (0%)	0 (0%)	0 (0%)	0 (0%)	0 (0%)	0 (0%)	0 (0%)
Other Milk Alternatives (n=88)	0 (0%)	0 (0%)	0 (0%)	0 (0%)	0 (0%)	0 (0%)	0 (0%)
**Energy Drinks (n=149)**	1 (1%)	0 (0%)	7 (5%)	17 (11%)	5 (3%)	13 (9%)	15 (10%)
**Hot Drinks (n=176)**	0 (0%)	0 (0%)	0 (0%)	0 (0%)	0 (0%)	0 (0%)	0 (0%)
Instant Coffee Mixes (n=19)	0 (0%)	0 (0%)	0 (0%)	0 (0%)	0 (0%)	0 (0%)	0 (0%)
Other Hot Drinks (n=157)	0 (0%)	0 (0%)	0 (0%)	0 (0%)	0 (0%)	0 (0%)	0 (0%)
**Juice (n=552)**	0 (0%)	8 (1%)	0 (0%)	29 (5%)	19 (3%)	1 (0%)	13 (2%)
Coconut Water (n=29)	0 (0%)	0 (0%)	0 (0%)	0 (0%)	0 (0%)	0 (0%)	0 (0%)
Juice Drinks and Nectars (n=523)	0 (0%)	8 (2%)	0 (0%)	29 (6%)	19 (4%)	1 (0%)	13 (2%)
**RTD Coffee (n=33)**	0 (0%)	0 (0%)	0 (0%)	0 (0%)	0 (0%)	0 (0%)	1 (3%)
**RTD Tea (n=358)**	8 (2%)	1 (0%)	0 (0%)	48 (13%)	7 (2%)	2 (1%)	45 (13%)
Carbonated RTD and Kombucha (n=25)	0 (0%)	0 (0%)	0 (0%)	8 (32%)	0 (0%)	0 (0%)	0 (0%)
Still RTD Tea (n=333)	8 (2%)	1 (0%)	0 (0%)	40 (12%)	7 (2%)	2 (1%)	45 (14%)
**Sports Drinks (n=149)**	10 (7%)	0 (0%)	0 (0%)	1 (1%)	0 (0%)	0 (0%)	0 (0%)

**Total (n=3386)**	**45 (1%)**	**58 (2%)**	**24 (1%)**	**113 (3%)**	**47 (1%)**	**26 (1%)**	**82 (2%)**
